# *o*-Phenyl­enediammonium bis­(3-carb­oxy-4-hydroxy­benzene­sulfonate)

**DOI:** 10.1107/S1600536809049071

**Published:** 2009-11-21

**Authors:** Yun-Sheng Ma, Wei-Wei Yang

**Affiliations:** a Department of Chemistry & Materials Engineering, Jiangsu Laboratory of Advanced Functional Materials, Changshu Institute of Technology, Changshu, 215500 Jiangsu, People’s Republic of China

## Abstract

In the title salt, C_6_H_10_N_2_^2+^·2C_7_H_5_O_6_S^−^, the negative charge of the anion resides on the sulfonate group. In the crystal, the cations and anions are ­linked by N—H⋯O and O—H⋯O hydrogen bonds, forming a three-dimensional network. The complete dication is generated by crystallographic twofold symmetry.

## Related literature

For related structures, see: Bakasova *et al.* (1991 ▶); Du *et al.* (2008 ▶); Meng *et al.* (2008 ▶); Raj *et al.* (2003 ▶); Smith (2005 ▶); Smith *et al.* (2004 ▶, 2005*a* ▶,*b* ▶,*c* ▶, 2006 ▶); Wang & Wei (2007 ▶).
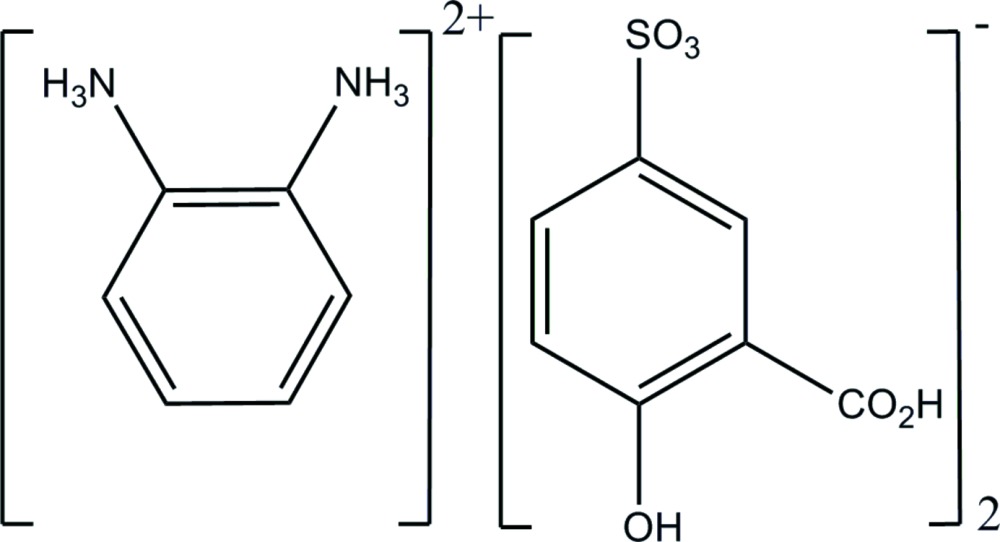


## Experimental

### 

#### Crystal data

C_6_H_10_N_2_^2+^·2C_7_H_5_O_6_S^−^
*M* *_r_* = 544.50
Monoclinic, 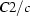

*a* = 11.667 (2) Å
*b* = 16.081 (3) Å
*c* = 12.356 (3) Å
β = 105.90 (3)°
*V* = 2229.5 (9) Å^3^
*Z* = 4
Mo *K*α radiation
μ = 0.31 mm^−1^
*T* = 566 K
0.30 × 0.28 × 0.24 mm

#### Data collection

Rigaku SCXmini diffractometer
Absorption correction: multi-scan (*CrystalClear*; Rigaku, 2005 ▶) *T*_min_ = 0.902, *T*_max_ = 0.923
11440 measured reflections
2543 independent reflections
2019 reflections with *I* > 2σ(*I*)
*R*_int_ = 0.042

#### Refinement

*R*[*F* ^2^ > 2σ(*F* ^2^)] = 0.048
*wR*(*F* ^2^) = 0.108
*S* = 1.00
2543 reflections
178 parameters
5 restraints
H atoms treated by a mixture of independent and constrained refinement
Δρ_max_ = 0.34 e Å^−3^
Δρ_min_ = −0.38 e Å^−3^

### 

Data collection: *CrystalClear* (Rigaku, 2005 ▶); cell refinement: *CrystalClear*; data reduction: *CrystalClear*; program(s) used to solve structure: *SHELXS97* (Sheldrick, 2008 ▶); program(s) used to refine structure: *SHELXL97* (Sheldrick, 2008 ▶); molecular graphics: *SHELXTL/PC* (Sheldrick, 2008 ▶); software used to prepare material for publication: *SHELXTL/PC* and *PLATON* (Spek, 2009 ▶).

## Supplementary Material

Crystal structure: contains datablocks I, global. DOI: 10.1107/S1600536809049071/ng2658sup1.cif

Structure factors: contains datablocks I. DOI: 10.1107/S1600536809049071/ng2658Isup2.hkl

Additional supplementary materials:  crystallographic information; 3D view; checkCIF report

## Figures and Tables

**Table 1:** Hydrogen-bond geometry (Å, °)

*D*—H⋯*A*	*D*—H	H⋯*A*	*D*⋯*A*	*D*—H⋯*A*
N1—H1*B*⋯O1^i^	0.856 (10)	2.46 (2)	3.157 (3)	139 (2)
N1—H1*B*⋯O4^i^	0.856 (10)	2.424 (18)	3.176 (3)	147 (2)
N1—H1*A*⋯O2^ii^	0.860 (10)	1.939 (11)	2.795 (3)	174 (3)
N1—H1*B*⋯O6^iii^	0.856 (10)	2.46 (3)	2.836 (3)	107 (2)
N1—H1*C*⋯O1	0.857 (10)	1.979 (17)	2.755 (3)	150 (3)
O3—H3*A*⋯O6	0.86 (3)	1.82 (2)	2.600 (3)	151 (3)
O5—H5*A*⋯O4^iv^	0.849 (10)	1.819 (10)	2.668 (2)	178 (3)

Symmetry codes: (i) 
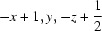
; (ii) 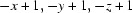; (iii) 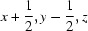; (iv) 
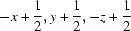
.

**Fig. 1.**
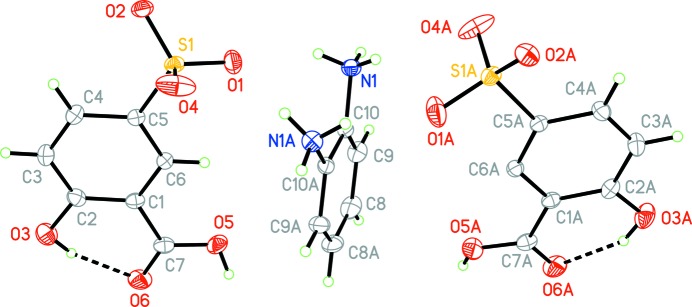
A view of the compound with the atomic numbering scheme. Displacement ellipsoids were drawn at the 30% probability level. [Symmetry code A = 1-x, y, 1/2-z]. The H-bonds are shown as dashed lines.

**Fig. 2.**
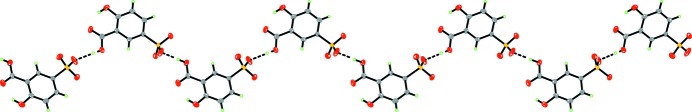
Viw of the one-dimensional hydrogen bonded chain of 3-carboxylato-4-hydroxybenzenesulfonate shown with dashed lines, running along the b axis.

**Fig. 3.**
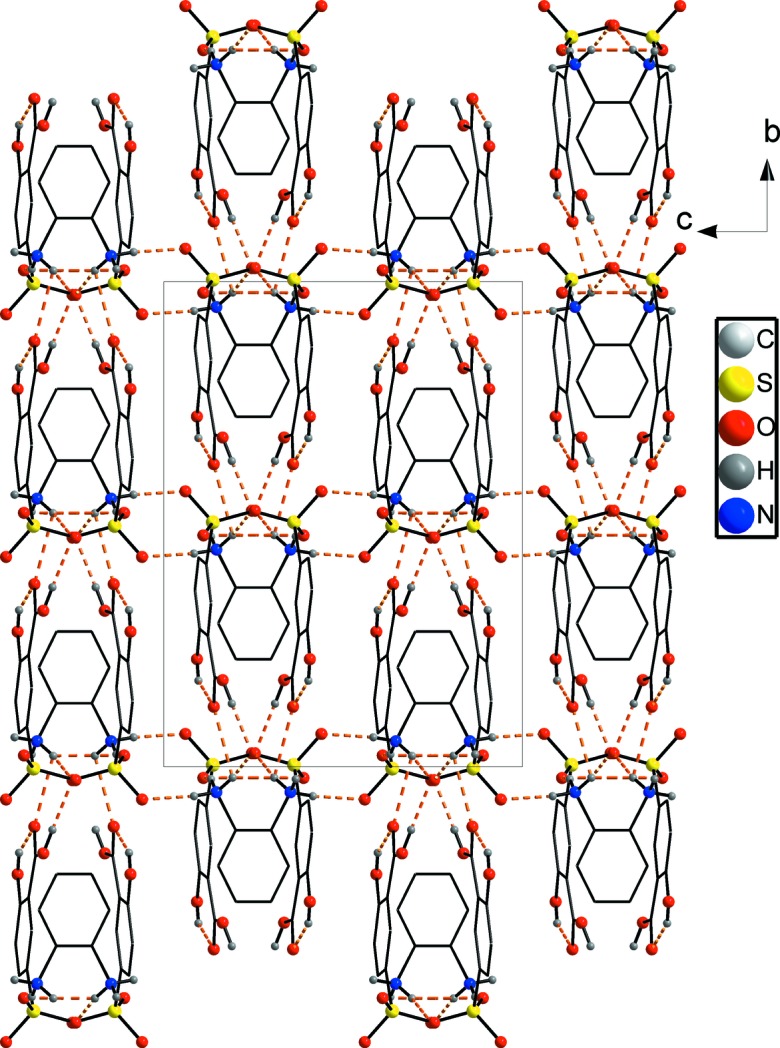
The cell packing diagram indicating hydrogen bonds links with dashed lines, viewed down the a axis.

## supplementary crystallographic information

### Comment

A number of proton-transfer compounds of 3-carboxy-4-hydroxybenzenesulfonic acid with Lewis bases have been widely studied because of the good crystallinity of many of the compounds (Smith *et al.*, 2004; Wang & Wei, 2007; Meng *et al.*, 2008; Du *et al.*, 2008). The feature is the presence of hydrogen-bonding interactions, resulted from the aminium donor group and the sulfonate and carboxyl O-atom acceptors. There are some aniline-type proton-transfer compounds reported during the past several years. These include compounds with aniline (Bakasova *et al.*, 1991), the 4-X-substituted anilines (X = F, Cl, Br) (Smith *et al.*, 2005b), 3-aminobenzoic acid (Smith 2005), 4-aminobenzoic acid (Smith *et al.*, 2005c), benzylamine (Smith *et al.*, 2006), 1,4-phenylenediamine (Smith *et al.*, 2005a). The present paper is concerned with the crystal structure of a new proton-transfer compound of 3-carboxy-4-hydroxybenzenesulfonic acid with 1,2-phenylenediamine as Lewis base.

In the compound, the asymmetric unit consists of one half 1,2-phenylenediaminium dication and one 3-carboxy-4-hydroxy-benzenesulfonate anion. The hydrogen atom was transferred from the sulfonic group to the amino nitrogen atom, forming an 1:2 organic salt. In the anion (Fig. 1 and Table 1), the carboxyl group is nearly coplanar with the benzene ring [the dihedral angle is 3.9 ^o^] and there is an intramolecular hydrogen bond O3—H···O6 2.600 (3) Å involving the hydroxy group and carboxyl atom. In addition, intermolecular hydrogen bonds between a sulfonate O atom and a carboxylate O atom [O5···O4 = 2.668 (2) Å; symmetry code: 1/2-x,1/2+y,1/2-z] connect the anions through head-to-tail into a one-dmensional chain (Fig. 2 and Table 2). The protonated N atoms form hydrogen bonds with three sulfonate O atoms and one carboxyl O atom [range 2.755 (3)—3.176 (3) Å) (Fig. 3 and Table 2), forming a three-dimensional network. The network are further consolidated by π—π stacking effects between the benzene rings of anions [the inter-ring centroid distance is 3.564 (1) Å].

### Experimental

The title compound was synthesized by heating 3-carboxy-4-hydroxybenzenesulfonic acid (0.218 g, 1 mmol) and 1,2-phenylenediamine (0.108 g, 1 mmol) in water (20 mL) for 2 hours. After evaporation the solution, dark-blue block crystals formed in high yield.

### Refinement

Carbon-bound H atoms were positioned geometrically (C—H = 0.93 Å), and were included in the refinement in the riding mode approximation, with *U*_iso_(H) = 1.2*U*_eq_(C). H atoms bound to O and N atoms were located in a difference Fourier map and refined with restraints [N—H and O—H = 0.85 (1) Å, with *U*_iso_(H) values fixed at 1.5*U*_eq_(N) and 1.5*U*_eq_(O)].

### Crystal data

**Table:**

C_6_H_10_N_2_^2+^·2C_7_H_5_O_6_S^−^	*F*(000) = 1128
*M**_r_* = 544.50	*D*_x_ = 1.622 Mg m^−^^3^
Monoclinic, *C*2/*c*	Mo *K*α radiation, λ = 0.71073 Å
Hall symbol: -C 2yc	Cell parameters from 6744 reflections
*a* = 11.667 (2) Å	θ = 3.3–27.6°
*b* = 16.081 (3) Å	µ = 0.31 mm^−^^1^
*c* = 12.356 (3) Å	*T* = 566 K
β = 105.90 (3)°	Block, dark-blue
*V* = 2229.5 (9) Å^3^	0.30 × 0.28 × 0.24 mm
*Z* = 4	

### Data collection

**Table:**

Rigaku SCXmini diffractometer	2543 independent reflections
Radiation source: fine-focus sealed tube	2019 reflections with *I* > 2σ(*I*)
graphite	*R*_int_ = 0.042
Detector resolution: 13.6612 pixels mm^-1^	θ_max_ = 27.5°, θ_min_ = 3.1°
dtfind.ref scans	*h* = −15→15
Absorption correction: multi-scan (*CrystalClear*; Rigaku, 2005)	*k* = −20→20
*T*_min_ = 0.902, *T*_max_ = 0.923	*l* = −15→15
11440 measured reflections	

### Refinement

**Table:**

Refinement on *F*^2^	Primary atom site location: structure-invariant direct methods
Least-squares matrix: full	Secondary atom site location: difference Fourier map
*R*[*F*^2^ > 2σ(*F*^2^)] = 0.048	Hydrogen site location: inferred from neighbouring sites
*wR*(*F*^2^) = 0.108	H atoms treated by a mixture of independent and constrained refinement
*S* = 1.00	*w* = 1/[σ^2^(*F*_o_^2^) + (0.0426*P*)^2^ + 3.7751*P*] where *P* = (*F*_o_^2^ + 2*F*_c_^2^)/3
2543 reflections	(Δ/σ)_max_ < 0.001
178 parameters	Δρ_max_ = 0.34 e Å^−^^3^
5 restraints	Δρ_min_ = −0.37 e Å^−^^3^

### Special details

**Table:**

Geometry. All esds (except the esd in the dihedral angle between two l.s. planes) are estimated using the full covariance matrix. The cell esds are taken into account individually in the estimation of esds in distances, angles and torsion angles; correlations between esds in cell parameters are only used when they are defined by crystal symmetry. An approximate (isotropic) treatment of cell esds is used for estimating esds involving l.s. planes.
Refinement. Refinement of F^2^ against ALL reflections. The weighted R-factor wR and goodness of fit S are based on F^2^, conventional R-factors R are based on F, with F set to zero for negative F^2^. The threshold expression of F^2^ > 2sigma(F^2^) is used only for calculating R-factors(gt) etc. and is not relevant to the choice of reflections for refinement. R-factors based on F^2^ are statistically about twice as large as those based on F, and R- factors based on ALL data will be even larger.

### Fractional atomic coordinates and isotropic or equivalent isotropic displacement parameters (Å^2^)

**Table:**

	*x*	*y*	*z*	*U*_iso_*/*U*_eq_	
C1	0.1315 (2)	0.73023 (13)	0.37688 (17)	0.0286 (5)	
C2	0.0224 (2)	0.71605 (15)	0.40005 (19)	0.0334 (5)	
C3	−0.0144 (2)	0.63524 (16)	0.4130 (2)	0.0400 (6)	
H3	−0.0870	0.6259	0.4285	0.048*	
C4	0.0566 (2)	0.56927 (14)	0.40305 (19)	0.0338 (5)	
H4	0.0314	0.5153	0.4108	0.041*	
C5	0.16661 (19)	0.58267 (13)	0.38137 (17)	0.0255 (4)	
C6	0.20342 (19)	0.66230 (13)	0.36823 (17)	0.0265 (4)	
H6	0.2765	0.6711	0.3535	0.032*	
C7	0.1671 (2)	0.81562 (14)	0.35783 (19)	0.0340 (5)	
C8	0.5460 (2)	0.77864 (15)	0.2988 (2)	0.0463 (7)	
H8	0.5770	0.8288	0.3315	0.056*	
C9	0.5925 (2)	0.70459 (15)	0.3483 (2)	0.0379 (6)	
H9	0.6545	0.7047	0.4142	0.046*	
C10	0.54644 (18)	0.63079 (13)	0.29932 (17)	0.0258 (4)	
S1	0.25193 (5)	0.49616 (3)	0.36307 (5)	0.03052 (16)	
O1	0.37475 (17)	0.52318 (12)	0.3896 (2)	0.0617 (6)	
O2	0.23442 (14)	0.43243 (10)	0.43955 (14)	0.0367 (4)	
O3	−0.05305 (18)	0.77829 (12)	0.40738 (17)	0.0513 (5)	
H3A	−0.014 (3)	0.8226 (13)	0.403 (3)	0.077*	
O4	0.2075 (2)	0.47241 (12)	0.24585 (15)	0.0656 (7)	
O5	0.27040 (17)	0.82146 (10)	0.33577 (16)	0.0444 (5)	
H5A	0.276 (3)	0.8699 (10)	0.310 (2)	0.067*	
O6	0.10406 (17)	0.87634 (10)	0.36053 (16)	0.0498 (5)	
N1	0.59386 (18)	0.55255 (12)	0.35330 (17)	0.0326 (4)	
H1A	0.6474 (19)	0.5606 (17)	0.4158 (14)	0.049*	
H1B	0.620 (2)	0.5226 (15)	0.3080 (19)	0.049*	
H1C	0.5367 (18)	0.5255 (15)	0.368 (2)	0.049*	

### Atomic displacement parameters (Å^2^)

**Table:**

	*U*^11^	*U*^22^	*U*^33^	*U*^12^	*U*^13^	*U*^23^
C1	0.0364 (12)	0.0250 (11)	0.0238 (10)	0.0015 (9)	0.0075 (9)	0.0000 (8)
C2	0.0353 (12)	0.0369 (13)	0.0288 (11)	0.0079 (10)	0.0101 (10)	0.0001 (10)
C3	0.0319 (13)	0.0456 (15)	0.0469 (14)	0.0002 (11)	0.0184 (11)	0.0031 (12)
C4	0.0349 (12)	0.0316 (12)	0.0364 (13)	−0.0060 (10)	0.0121 (10)	0.0003 (10)
C5	0.0297 (11)	0.0241 (10)	0.0233 (10)	0.0008 (8)	0.0080 (9)	−0.0009 (8)
C6	0.0278 (11)	0.0261 (11)	0.0258 (11)	−0.0005 (9)	0.0079 (9)	−0.0008 (8)
C7	0.0448 (14)	0.0277 (12)	0.0280 (12)	0.0027 (10)	0.0073 (10)	−0.0003 (9)
C8	0.0600 (18)	0.0249 (12)	0.0618 (17)	−0.0104 (11)	0.0298 (13)	−0.0116 (11)
C9	0.0422 (14)	0.0343 (13)	0.0382 (13)	−0.0092 (11)	0.0126 (11)	−0.0088 (10)
C10	0.0263 (11)	0.0235 (10)	0.0288 (11)	−0.0002 (8)	0.0094 (9)	0.0011 (8)
S1	0.0387 (3)	0.0218 (3)	0.0332 (3)	0.0037 (2)	0.0134 (2)	0.0026 (2)
O1	0.0403 (11)	0.0380 (11)	0.1180 (19)	0.0047 (8)	0.0410 (12)	0.0108 (11)
O2	0.0404 (9)	0.0279 (8)	0.0394 (9)	−0.0004 (7)	0.0070 (7)	0.0091 (7)
O3	0.0508 (12)	0.0455 (11)	0.0653 (12)	0.0171 (9)	0.0291 (10)	0.0021 (10)
O4	0.1183 (19)	0.0432 (11)	0.0312 (10)	0.0353 (12)	0.0134 (11)	−0.0044 (8)
O5	0.0530 (11)	0.0263 (9)	0.0593 (12)	−0.0020 (8)	0.0246 (9)	0.0068 (8)
O6	0.0623 (12)	0.0272 (9)	0.0616 (12)	0.0110 (8)	0.0200 (10)	0.0001 (8)
N1	0.0339 (11)	0.0304 (11)	0.0322 (11)	0.0019 (9)	0.0070 (8)	0.0050 (9)

### Geometric parameters (Å, °)

**Table:**

C1—C2	1.398 (3)	C8—C9	1.380 (4)
C1—C6	1.399 (3)	C8—H8	0.9300
C1—C7	1.472 (3)	C9—C10	1.373 (3)
C2—O3	1.352 (3)	C9—H9	0.9300
C2—C3	1.391 (3)	C10—C10^i^	1.391 (4)
C3—C4	1.372 (3)	C10—N1	1.460 (3)
C3—H3	0.9300	S1—O2	1.4459 (16)
C4—C5	1.398 (3)	S1—O1	1.447 (2)
C4—H4	0.9300	S1—O4	1.4495 (19)
C5—C6	1.374 (3)	O3—H3A	0.86 (3)
C5—S1	1.761 (2)	O5—H5A	0.849 (10)
C6—H6	0.9300	N1—H1A	0.860 (10)
C7—O6	1.228 (3)	N1—H1B	0.856 (10)
C7—O5	1.310 (3)	N1—H1C	0.857 (10)
C8—C8^i^	1.378 (6)		
			
C2—C1—C6	119.2 (2)	C8^i^—C8—H8	119.8
C2—C1—C7	119.8 (2)	C9—C8—H8	119.8
C6—C1—C7	121.0 (2)	C10—C9—C8	119.5 (2)
O3—C2—C3	117.2 (2)	C10—C9—H9	120.3
O3—C2—C1	122.6 (2)	C8—C9—H9	120.3
C3—C2—C1	120.2 (2)	C9—C10—C10^i^	120.17 (14)
C4—C3—C2	119.9 (2)	C9—C10—N1	119.4 (2)
C4—C3—H3	120.0	C10^i^—C10—N1	120.45 (11)
C2—C3—H3	120.0	O2—S1—O1	111.92 (12)
C3—C4—C5	120.4 (2)	O2—S1—O4	112.98 (12)
C3—C4—H4	119.8	O1—S1—O4	111.50 (15)
C5—C4—H4	119.8	O2—S1—C5	106.86 (10)
C6—C5—C4	120.0 (2)	O1—S1—C5	107.06 (11)
C6—C5—S1	121.00 (17)	O4—S1—C5	106.05 (11)
C4—C5—S1	118.93 (16)	C2—O3—H3A	104 (2)
C5—C6—C1	120.3 (2)	C7—O5—H5A	108 (2)
C5—C6—H6	119.8	C10—N1—H1A	111.8 (19)
C1—C6—H6	119.8	C10—N1—H1B	110.2 (19)
O6—C7—O5	122.7 (2)	H1A—N1—H1B	112 (3)
O6—C7—C1	122.7 (2)	C10—N1—H1C	108.4 (19)
O5—C7—C1	114.6 (2)	H1A—N1—H1C	107 (3)
C8^i^—C8—C9	120.33 (15)	H1B—N1—H1C	107 (3)
			
C6—C1—C2—O3	−178.6 (2)	C2—C1—C7—O6	−0.5 (3)
C7—C1—C2—O3	−0.9 (3)	C6—C1—C7—O6	177.2 (2)
C6—C1—C2—C3	−0.7 (3)	C2—C1—C7—O5	−179.4 (2)
C7—C1—C2—C3	177.0 (2)	C6—C1—C7—O5	−1.7 (3)
O3—C2—C3—C4	178.0 (2)	C8^i^—C8—C9—C10	0.1 (5)
C1—C2—C3—C4	0.0 (4)	C8—C9—C10—C10^i^	−0.1 (4)
C2—C3—C4—C5	0.9 (4)	C8—C9—C10—N1	−178.5 (2)
C3—C4—C5—C6	−1.0 (3)	C6—C5—S1—O2	147.63 (18)
C3—C4—C5—S1	−177.25 (19)	C4—C5—S1—O2	−36.1 (2)
C4—C5—C6—C1	0.2 (3)	C6—C5—S1—O1	27.6 (2)
S1—C5—C6—C1	176.44 (16)	C4—C5—S1—O1	−156.21 (19)
C2—C1—C6—C5	0.6 (3)	C6—C5—S1—O4	−91.6 (2)
C7—C1—C6—C5	−177.1 (2)	C4—C5—S1—O4	84.6 (2)

Symmetry codes: (i) −*x*+1, *y*, −*z*+1/2.

### Hydrogen-bond geometry (Å, °)

**Table:**

*D*—H···*A*	*D*—H	H···*A*	*D*···*A*	*D*—H···*A*
N1—H1B···O1^i^	0.86 (1)	2.46 (2)	3.157 (3)	139 (2)
N1—H1B···O4^i^	0.86 (1)	2.42 (2)	3.176 (3)	147 (2)
N1—H1A···O2^ii^	0.86 (1)	1.94 (1)	2.795 (3)	174 (3)
N1—H1B···O6^iii^	0.86 (1)	2.46 (3)	2.836 (3)	107 (2)
N1—H1C···O1	0.86 (1)	1.98 (2)	2.755 (3)	150 (3)
O3—H3A···O6	0.86 (3)	1.82 (2)	2.600 (3)	151 (3)
O5—H5A···O4^iv^	0.85 (1)	1.82 (1)	2.668 (2)	178 (3)

Symmetry codes: (i) −*x*+1, *y*, −*z*+1/2; (ii) −*x*+1, −*y*+1, −*z*+1; (iii) *x*+1/2, *y*−1/2, *z*; (iv) −*x*+1/2, *y*+1/2, −*z*+1/2.
